# Japanese Encephalitis Virus (JEV) NS1′ Enhances the Viral Infection of Dendritic Cells (DCs) and Macrophages in Pig Tonsils

**DOI:** 10.1128/spectrum.01147-22

**Published:** 2022-06-22

**Authors:** Shengda Xie, Junhui Pan, Qiuting Zhang, Ruting Guan, Xingmiao Yang, Xiaoxiao Yang, Zhenjie Liang, Ruibing Cao

**Affiliations:** a MOE Joint International Research Laboratory of Animal Health and Food Safety, College of Veterinary Medicine, Nanjing Agricultural University, Nanjing, China; Center for Research and Advanced Studies (CINVESTAV-IPN)

**Keywords:** JEV, NS1′, pig, tonsil, infection

## Abstract

Pigs are the amplifying hosts of Japanese encephalitis virus (JEV). Currently, the safe and effective live attenuated vaccine made of JEV strain SA14-14-2, which does not express NS1′, is widely used in humans and domestic animals to prevent JEV infection. In this study, we constructed the NS1′ expression recombinant virus (rA66G) through a single nucleotide mutation in *NS2A* of JEV strain SA14-14-2. Animal experiments showed that NS1′ significantly enhanced JEV infection in pig central nervous system (CNS) and tonsil tissues. Pigs shed virus in oronasal secretions in the JEV rA66G virus inoculation group, indicating that NS1′ may facilitate the horizontal transmission of JEV. Additionally, dendritic cells (DCs) and macrophages are the main target cells of JEV infection in pig tonsils, which are an important site of persistent JEV infection. The reduction of major histocompatibility complex class II (MHC II) and activation of inducible nitric oxide synthase (iNOS) in pig tonsils caused by viral infection may create a beneficial environment for persistent JEV infection. These results are of significance for JEV infection in pigs and lay the foundation for future studies of JEV persistent infection in pig tonsils.

**IMPORTANCE** Pigs are amplification hosts for Japanese encephalitis virus (JEV). JEV can persist in the tonsils for months despite the presence of neutralizing antibodies. The present study shows that NS1′ increases JEV infection in pig tonsils. In addition, DCs and macrophages in the tonsils are the target cells for JEV infection, and JEV NS1′ promotes virus infection in DCs and macrophages. This study reveals a novel function of JEV NS1′ protein and lays the foundation for future studies of JEV persistent infection in pig tonsils.

## INTRODUCTION

The mosquito-borne Japanese encephalitis virus (JEV) is an important zoonotic pathogen that was first isolated from a human in Japan in 1935 (1, 2). JEV, which belongs to the family of *Flaviviridae*, is the main pathogen causing viral encephalitis. As an arthropod-borne virus, JEV transmits between mosquito vectors and vertebrate hosts, especially pigs and wading birds (3). Humans are dead-end hosts of JEV, while pigs are amplification hosts of this virus (4, 5). Despite the generally asymptomatic nature of infection in commercial pigs, JEV infection in pregnant sows mainly manifests as reproductive diseases such as abortion and stillbirth, which can become important agricultural and food security issues (6). After JEV infection, pigs exhibit high levels of viremia, and the virus can persist in the tonsils for months (7, 8). Vector-free transmission of JEV between pigs has recently been described (7). Although this phenomenon has not been verified under natural conditions, persistently infected pigs could discharge virus under certain conditions (e.g., stress response) and thus infect other healthy pigs by vector-free transmission.

NS1′, a 52-amino acid C-terminal extension of NS1 protein, is produced as a result of −1 programmed ribosomal frameshifting (–1PRF) at the conserved slippery heptanucleotide (YCCUUUU) and 3′-adjacent pseudoknot near the beginning of the *NS2A* coding gene (9, 10). Consistent with NS1, NS1′ protein has three forms, a monomer, dimer (membrane-bound protein), and hexamer (secreted protein) (11–13). Flavivirus NS1 protein contains two conserved N-linked glycans at Asn-130 (N130) and Asn-207 (N207). N-glycosylation at these sites is critical for the biological function of NS1 (14–16). The glycosylation sites of NS1′ are inferred from NS1 protein, and no study has yet reported that 52 frameshift amino acids in NS1′ protein undergo glycosylation. NS1′ protein was reported to inhibit type I interferon (IFN) production and promote viral replication in host cells (17, 18). Other evidence demonstrated that abolishing NS1′ in JEV resulted in the attenuation of viral neuroinvasiveness and neurovirulence in mice (19). However, whether NS1′ enhances JEV infection in pigs is still unknown. Additionally, NS1′ enhances the viral multiplication in the peripheral tissue in mice (18). Tonsils are the main site of JEV replication in pigs. Mice, lacking tonsils, cannot be used as a suitable animal model, while pigs are relatively difficult to study as an animal model. Therefore, it is unclear whether NS1′ promotes JEV replication in pig tonsils.

In this study, we focused on two scientific questions: the roles of NS1′ in virus replication in pigs and the cell tropism of JEV in pig tonsils. Using reverse genetic techniques and animal models, we showed that NS1′ increased viral burden in pig tonsils. In addition, we found that the main target cells of JEV infection in the tonsils were dendritic cells (DCs) and macrophages.

## RESULTS

### A single nucleotide mutation in the *NS2A* gene (A66G) of JEV strain SA14-14-2 results in the expression of NS1′.

**FIG 1:**
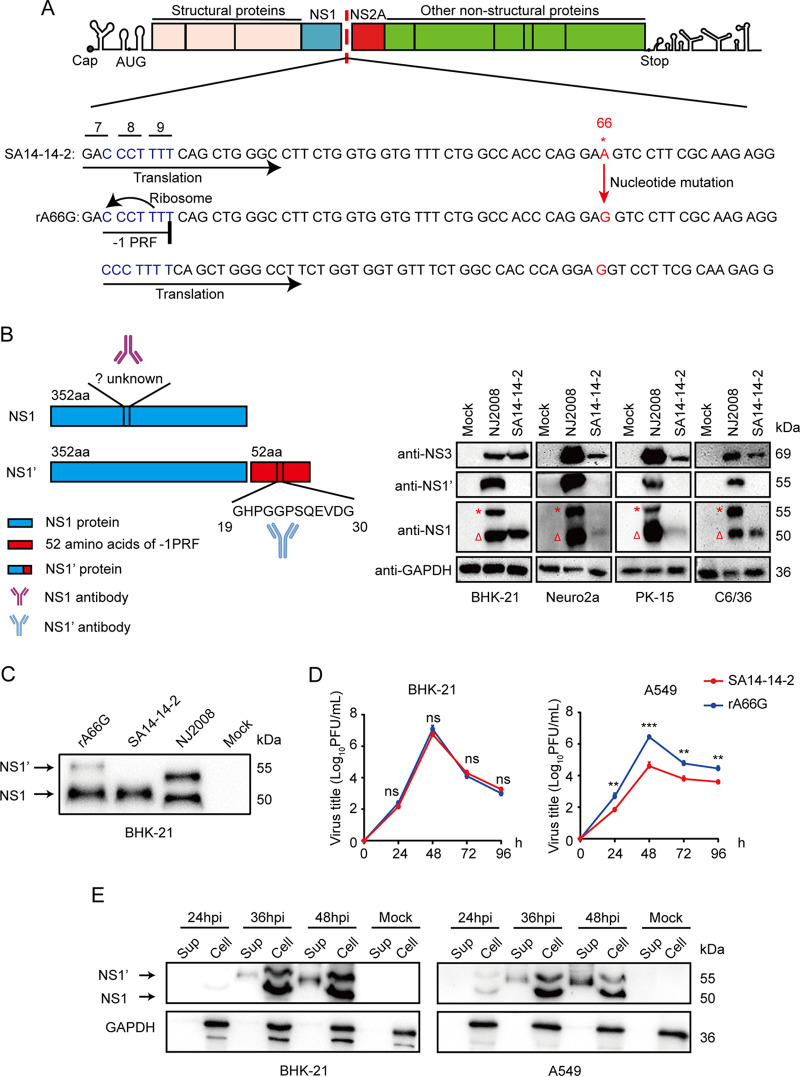
Construction and characterization of the A66G mutant in the *NS2A* gene of JEV strain SA14-14-2. (A) Schematic diagram of −1 programmed ribosomal frameshift (–PRF) and rA66G mutant virus construction. The red arrow represents a single nucleotide mutation. (B) Schematic diagram of epitopes recognized by NS1 and NS1′ monoclonal antibodies (left). BHK-21, Neuro2a, PK-15, and C6/36 cells were infected with JEV NJ2008 (expressing NS1′) or SA14-14-2 (not expressing NS1′) strain for 24 h; the specificity of our generated anti-NS1 and NS1′ antibodies was detected by Western blotting (right). *, the NS1′ band, Δ, the NS1 band. (C) BHK-21 cells were inoculated with JEV SA14-14-2, NJ2008, and rA66G mutant virus for 24 h. The expression of NS1 and NS1′ was detected by Western blotting with our generated anti-NS1 or NS1′ antibodies. JEV strain SA14-14-2 was used as a negative control, and JEV strain NJ2008 as a positive control. Multiplicity of infection (MOI) = 1. (D) Growth curves of rA66G virus on BHK-21 and A549 cells. The cultured supernatants were harvested at the indicated time points to estimate the titer of progeny by plaque assays using Vero cells. Data are presented as the mean ± standard deviation (SD) from three independent experiments. ns, not significant; **, *P < *0.01; ***, *P < *0.001. (E) BHK-21 or A549 cells were infected with JEV rA66G mutant virus for indicated hours. NS1 and NS1’ proteins secreted into the supernatant were detected by WB. MOI = 1. Sup, supernatant. WB, Western blotting.

It is known that nucleotide 66 of the *NS2A* gene is essential for NS1′ expression. In the NS2A-coding sequence of SA14-14-2 (not expressing NS1′), the 66th nucleotide is adenine (A), while its parental strain SA14 (expressing NS1′) has guanine (G) at the same position (19). Compared to its parental SA14 strain, the single-nucleotide difference (66A) in the *NS2A* gene of SA14-14-2 virus significantly shortens the length of stem 1 and destabilizes the pseudoknot structure and therefore cannot express NS1′ (19). In order to study the NS1′ protein, the A66G mutation in the *NS2A* gene was introduced into the infectious cDNA clone of JEV strain SA14-14-2 by the site-directed mutagenesis (Fig. 1A). Due to the genetic code degeneracy (both GAA and GAG encode glutamate), mutation of a single nucleotide does not alter the amino acid sequence of NS2A. In addition, we generated monoclonal antibodies against JEV NS1 and NS1′ proteins with very good specificity to detect the infection of rA66G (Fig. 1B). BHK-21 cells were infected with rA66G virus and the parental JEV SA14-14-2 strain to verify the expression of NS1′. Western blotting results revealed that rA66G virus and virulent JEV strain NJ2008 consistently expressed both NS1 and NS1′, whereas JEV strain SA14-14-2 only expressed NS1 (Fig. 1C). In BHK-21 cells, rA66G virus replicated as efficiently as JEV strain SA14-14-2, however, in the interferon-sensitive cell line (A549), the multiplication of JEV strain SA14-14-2 was somewhat reduced compared to the rA66G virus (Fig. 1D), which was consistent with previous studies (18). Similarly, the NS1′ protein expressed by rA66G virus was also secreted into the supernatants (Fig. 1E). These results indicated that the rA66G mutant virus was successfully constructed.

### NS1′ increases the infection of JEV in pigs.

**FIG 2:**
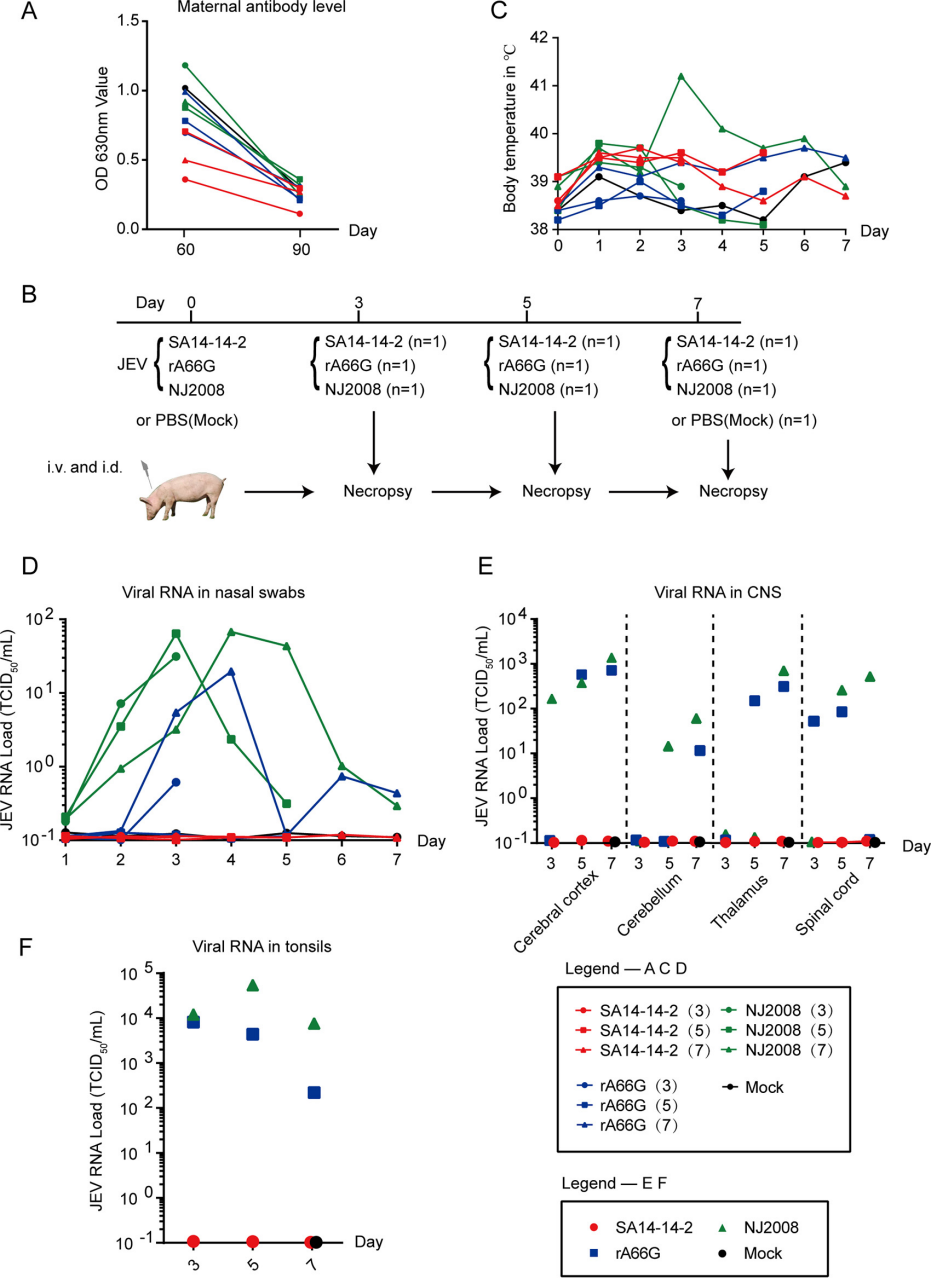
Body temperature and viral RNA loads in CNS and tonsil tissues of pigs. (A) Maternal antibodies against JEV in 10 pigs were detected by the ELISA. (B) Schematic diagram of JEV inoculation in pigs. (C) The daily body temperature of 10 pigs after JEV inoculation. (D) Nasal shedding of JEV from infected pigs was quantified by qRT-PCR. (E and F) JEV RNA loads were detected by qRT-PCR in the CNS (E) and tonsil (F) tissues. Each dot represents one animal. qRT-PCR, quantitative reverse transcription polymerase chain reaction.

A total of 10 healthy 60-day-old large white pigs (5 castrated males and 5 females) were selected for this study. Before inoculation, all pigs were healthy and alert, with body temperatures of 38.6 to 39.5°C and maternal antibodies against JEV decreased to low levels (Fig. 2A). Pigs were infected with 2 × 10^7^ 50% tissue culture infective dose (TCID_50_) of JEV SA14-14-2 (*n* = 3), NJ2008 (*n* = 3), or rA66G (*n* = 3) virus in a volume of 2 mL, giving one-half of the dose intravenously (i.v.) and the other half intradermally (i.d.) in the neck region. The remaining pig was injected with phosphate-buffered saline (PBS) as a control (*n* = 1). One pig from each JEV-inoculated group was euthanized on days 3, 5, and 7 postinfection, and the control pig was euthanized on day 7 (Fig. 2B). Only one JEV-infected pig reached a body temperature above 41°C; the other pigs showed a slight increase in body temperature (Fig. 2C). To characterize the nasal shedding dynamics of JEV in pigs, secretions from the nose were collected daily from alternating nares. Quantitative reverse transcriptase PCR (qRT-PCR) was used to test viral loading (Fig. 2D). No viral load was detected in the SA14-14-2 virus inoculation group throughout the experimental period. Two rA66G virus-infected and three NJ2008 virus-infected pigs started shedding virus oronasally as early as 2 days postinfection. Similarly, central nervous system (CNS) and tonsil tissues remained negative throughout the observation period in the SA14-14-2 virus inoculation group (Fig. 2E and F). Most of the tested areas of the CNS and tonsil tissues contained viral RNA from day 3 to day 7 postinfection in the rA66G and NJ2008 virus inoculation groups (Fig. 2E and F). Consistent with previous studies (8), the highest viral load was found in the tonsils, approximately 1 × 10^4^ TCID_50_/mL.

### NS1′ enhances the neuroinvasiveness of JEV in pigs.

**FIG 3:**
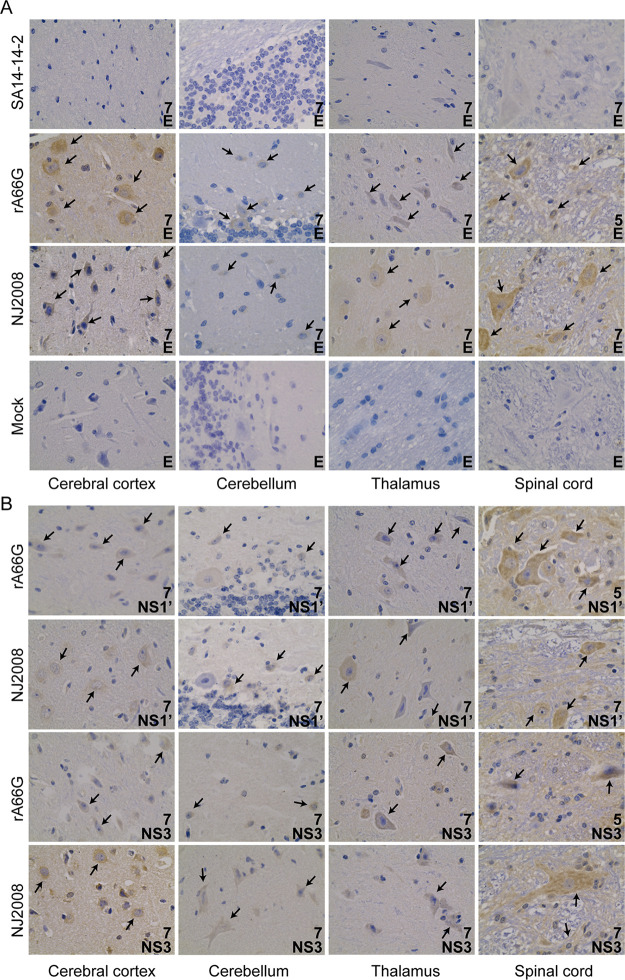
NS1′ enhanced JEV infection in CNS tissues. (A and B) CNS tissues (cerebral cortex, cerebellum, thalamus, and spinal cord) were collected at 5 dpi or 7 dpi and sectioned into 5-μm slides. JEV E (A), NS1′, or NS3 (B) antigen was demonstrated by IHC staining. The numbers in the lower right corner represent days postinfection. Arrows indicate JEV antigen-positive cells. Magnification, ×40. IHC, immunohistochemistry.

To investigate the role of NS1′ in JEV infection of the pig, we performed immunohistochemistry (IHC) and hematoxylin and eosin (H&E) staining on JEV-positive CNS tissues. IHC results showed that no JEV E antigen-positive cells were detected in the cerebral cortex, cerebellum, thalamus, and spinal cord of pigs inoculated with SA14-14-2 virus, which was similar to the mock control group (Fig. 3A). In the rA66G and NJ2008 virus inoculation groups, JEV-positive cells were observed in the cerebral cortex, cerebellum, thalamus, and spinal cord, and no statistically significant difference was observed between these two groups (Fig. 3A). JEV mainly infected neurons that were polygonal or triangular, such as pyramidal cells in the cerebral cortex, Purkinje cells, and basket cells in the cerebellum (although relatively few JEV-positive cells in the cerebellum) (Fig. 3A). In addition, CNS tissues collected from the rA66G and NJ2008 virus inoculation groups were subjected to immunohistochemistry with anti-NS1′ and NS3 antibodies. Similar to the anti-E results, neurons were the main target cells for JEV infection in pigs (Fig. 3B).

**FIG 4:**
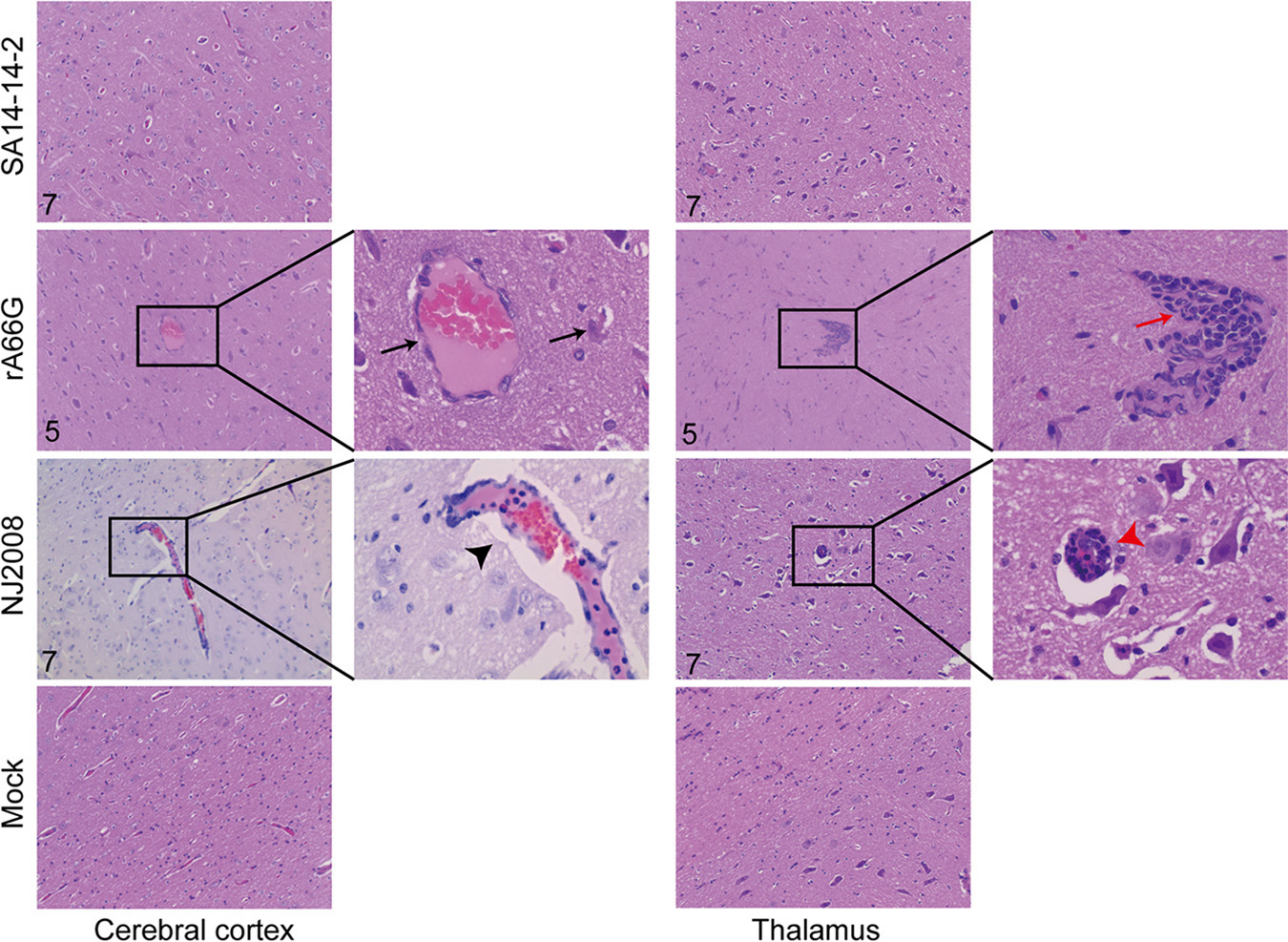
JEV caused neuronal damage in pig brains. Neuropathology in the cerebral cortex and thalamus of the JEV-infected pig. Black arrows indicate neuronal degeneration or necrosis and parenchymal infiltration by inflammatory cells. The black arrowhead indicates perivascular cuffing with lymphocytes, glial cell aggregates, neuronal degeneration, and necrosis with focal neuronophagia. The red arrow indicates multifocal glial nodules. The red arrowhead indicates the satellite phenomenon. Magnification, ×10 and ×40. Slides were analyzed by an independent pathologist in a blind manner.

Because of the relatively low viral load in the cerebellum, we focused on the pathological features of the cerebral cortex and thalamus caused by JEV. In both the rA66G and NJ2008 virus inoculation groups, conspicuous lesions in brains were observed. These lesions included perivascular cuffing with lymphocytes, multifocal gliosis, and neuronal degeneration and necrosis, all of which were characteristic of nonsuppurative encephalitis. Samples from the rA66G virus inoculation group had significant neuronal degeneration or necrosis and parenchymal infiltration by inflammatory cells within the cerebral cortex. Multifocal glial nodules were present in the thalamus and accompanied by mononuclear perivascular cuffs (Fig. 4). In the NJ2008 virus inoculation group, there was nonsuppurative encephalitis in the cerebral cortex, which was characterized by perivascular cuffing with lymphocytes, glial cell aggregates, neuronal degeneration, and necrosis with focal neuronophagia. In the thalamus, a satellite phenomenon was also observed in which a larger lesion was surrounded by smaller lesions (Fig. 4).

### NS1′ increased JEV infection in tonsils.

**FIG 5:**
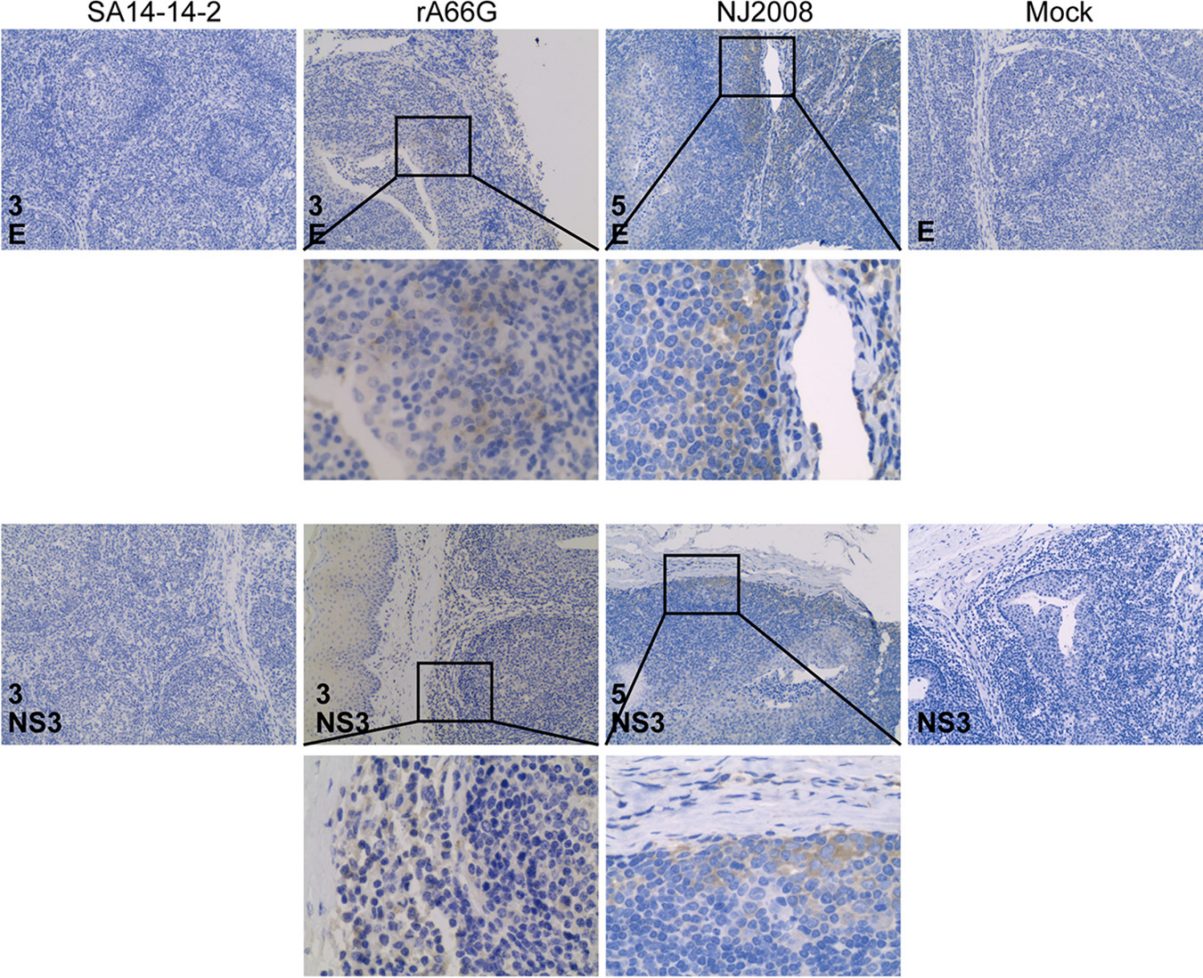
NS1′ enhanced JEV infection in pig tonsils. Tonsils were collected at 3 dpi or 5 dpi and sectioned into 5-μm slides. JEV E and NS3 antigens were demonstrated by IHC staining. JEV antigen-positive cells were mainly concentrated in the follicles. Magnification, ×10 and ×40. IHC, immunohistochemistry.

Tonsils, as an immune organ, are the primary peripheral replication site of JEV, and JEV can persist in tonsils for at least 30 days despite the presence of neutralizing antibodies (7, 20). Pigs have five tonsils, of which the palatine tonsil is the best developed and most widely noticed (21). A large number of JEV antigen-positive cells were present in margins of lymph follicle of the palatine tonsil; such cells had large nuclei with abundant cytoplasm and resembled macrophages (Fig. 5).

**FIG 6:**
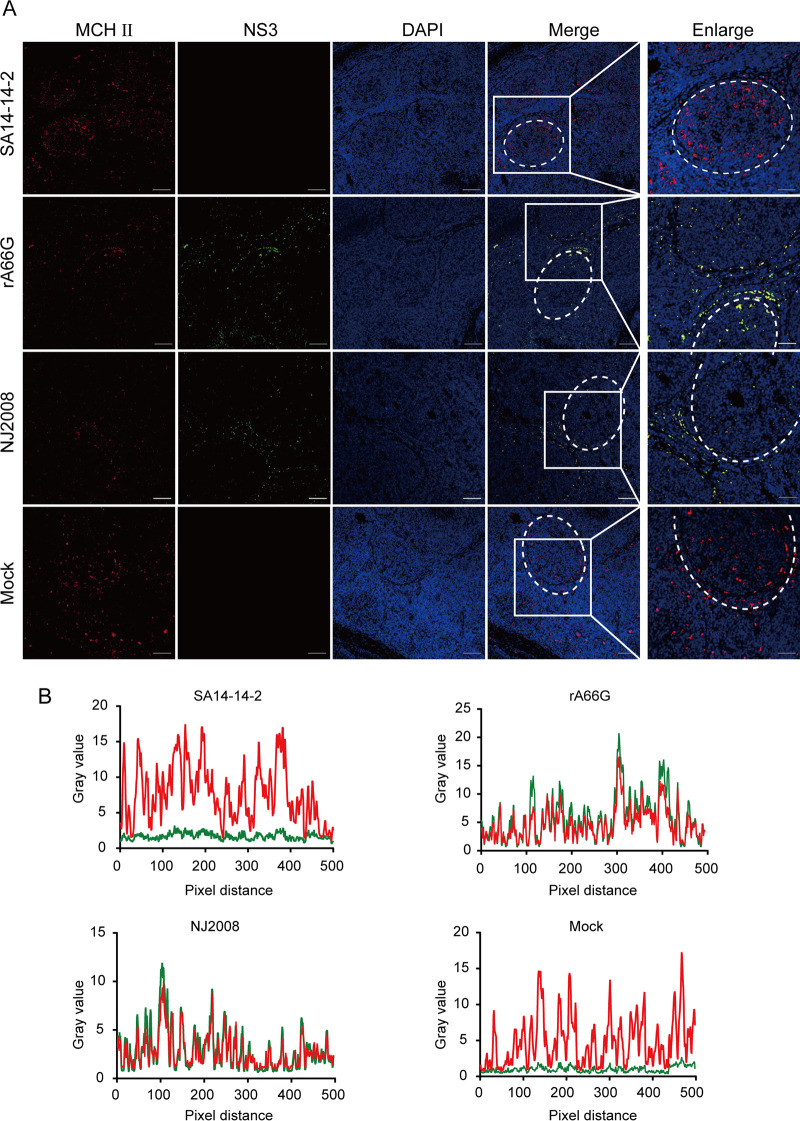
JEV antigen colocated with MHC II-positive cells in pig tonsils. (A) Tonsils were sectioned into 5-μm slides and subjected to coimmunofluorescence staining with anti-MHC II antibody [red] and anti-JEV NS3 antibody [green]). Nuclei were stained with DAPI (blue). White dotted circles represent the follicles. Magnification, ×10 and ×40. (B) The colocalization of MHC II and JEV antigen was analyzed with ImageJ software. Representative immunofluorescence images and colocalization line graphs of three independent experiments are shown.

When sucking blood, *Culex*, *Aedes*, and *Anopheles* mosquitos deposit JEV in the epidermis and dermis of the bitten host, where the viruses encounter cells that allow infection (22, 23). Intradermal fibroblasts and immune cells (DCs and macrophages) are considered to be the main cells for the initial arboviral replication (24). Next, infected DCs migrate to draining lymph nodes, where a second round of viral replication takes place, and then the progeny viruses enter the circulatory system to form viremia (24, 25). Owing to the importance of DCs and macrophages in the systemic dissemination of JEV in mammals, we used histocompatibility complex class II (MHC II) as a cellular marker for DCs and macrophages to explore whether JEV primarily infects these cells in pig tonsils. The stained tissues were analyzed using immunofluorescence. In the SA14-14-2 virus inoculation group, JEV antigen-positive cells were not observed. MCH II-positive cells were mainly found in lymphoid follicles (Fig. 6A and B). However, in the rA66G virus inoculation group, DCs and macrophages were positive for the JEV NS3 antigen (Fig. 6A and B). After rA66G or NJ2008 virus infection, DCs and macrophages migrated from the center of the lymphoid follicles to the periphery and even to the interfollicular region (Fig. 6A). Additionally, a significant reduction in MHC II molecule was observed in rA66G or NJ2008 virus-infected tonsils. These results demonstrated that DCs and macrophages may be the main target cells of JEV infection in pig tonsils.

**FIG 7:**
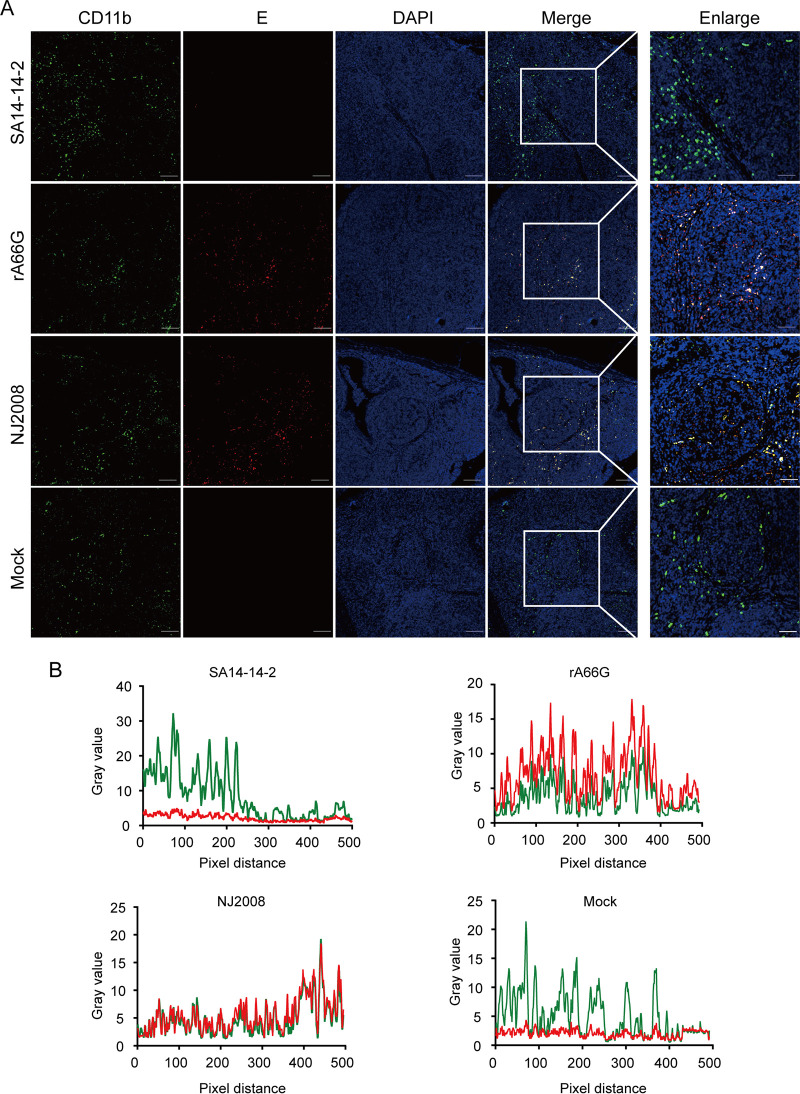
JEV antigen colocated with CD11b-positive cells. (A) Tonsils were sectioned into 5 μm slides and stained for anti-CD11b antibody (green) and anti-JEV E antibody (red). Nuclei were stained with DAPI (blue). Magnification, ×10 and ×40. (B) The colocalization of CD11b and JEV antigen was analyzed with ImageJ software. Representative immunofluorescence images and colocalization line graphs of three independent experiments are shown.

**FIG 8:**
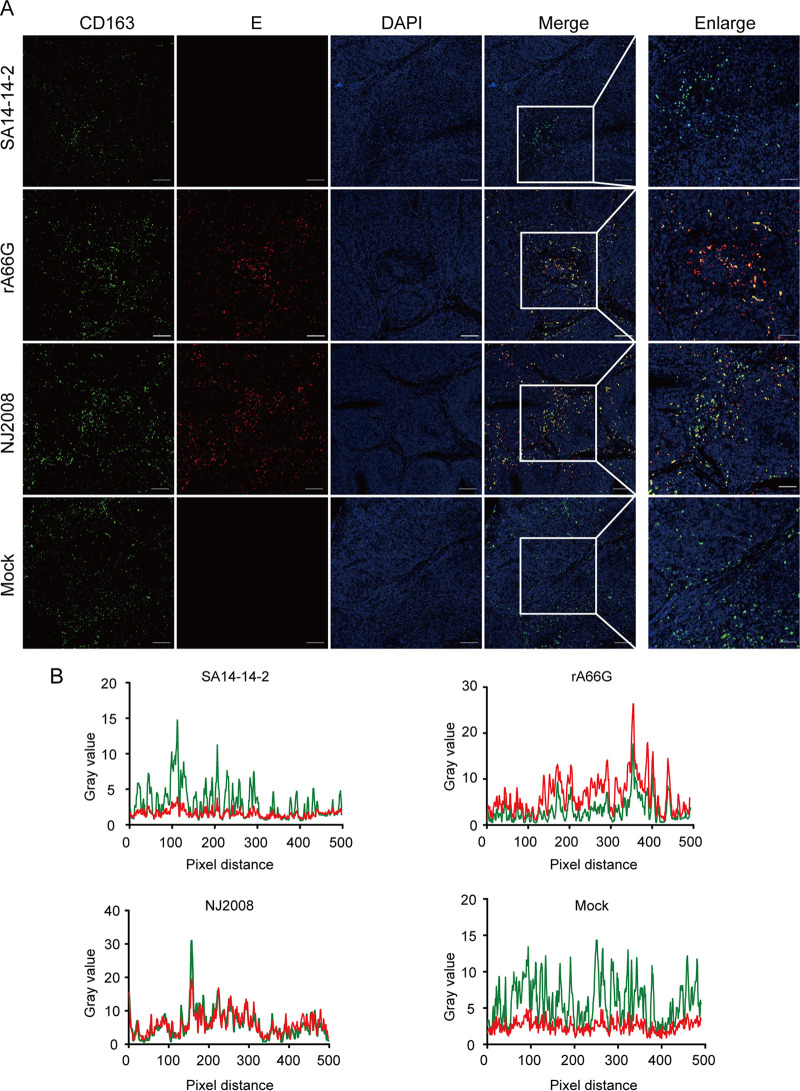
JEV antigen colocated with CD163-positive cells. (A) Tonsils were sectioned into 5 μm slides and subjected to coimmunofluorescence staining with anti-CD163 antibody (green) and anti-JEV E antibody (red). Nuclei were stained with DAPI (blue). Magnification, ×10 and ×40. (B) The colocalization of CD163 and JEV antigen was analyzed with ImageJ software. Representative immunofluorescence images and colocalization line graphs of three independent experiments a shown.

To exclude the interference from MHC II-expressing B cells, we stained tissue sections with antibodies against JEV E and CD11b (pig DC marker) and CD163 (pig macrophage marker), respectively. The cell tropism of rA66G virus was consistent with those of JEV virulent strain NJ2008. JEV E protein colocalized with either CD11b or CD163, and the double positive cells were also mainly located around the lymphoid follicles (Fig. 7A and B, Fig. 8A and B). However, compared to the mock group and the SA14-14-2 virus inoculation group, the reduction of CD163 and CD11b was not observed in the rA66G and NJ2008 virus inoculation groups. These results suggested that this phenomenon may be caused by the downregulated expression of MHC II molecules, rather than a decrease in the number of DCs and macrophages. The low-level expression of MHC II diminishes the antigen-presenting ability of antigen-presenting cells (APCs), which inhibits T and B cell activation, resulting in immune exemption or immune tolerance (26). This may be one of the reasons why JEV can cause persistent infection in pig tonsils.

### cDC2 cells are the preferred cell subtype of JEV infection in pig tonsils.

**FIG 9:**
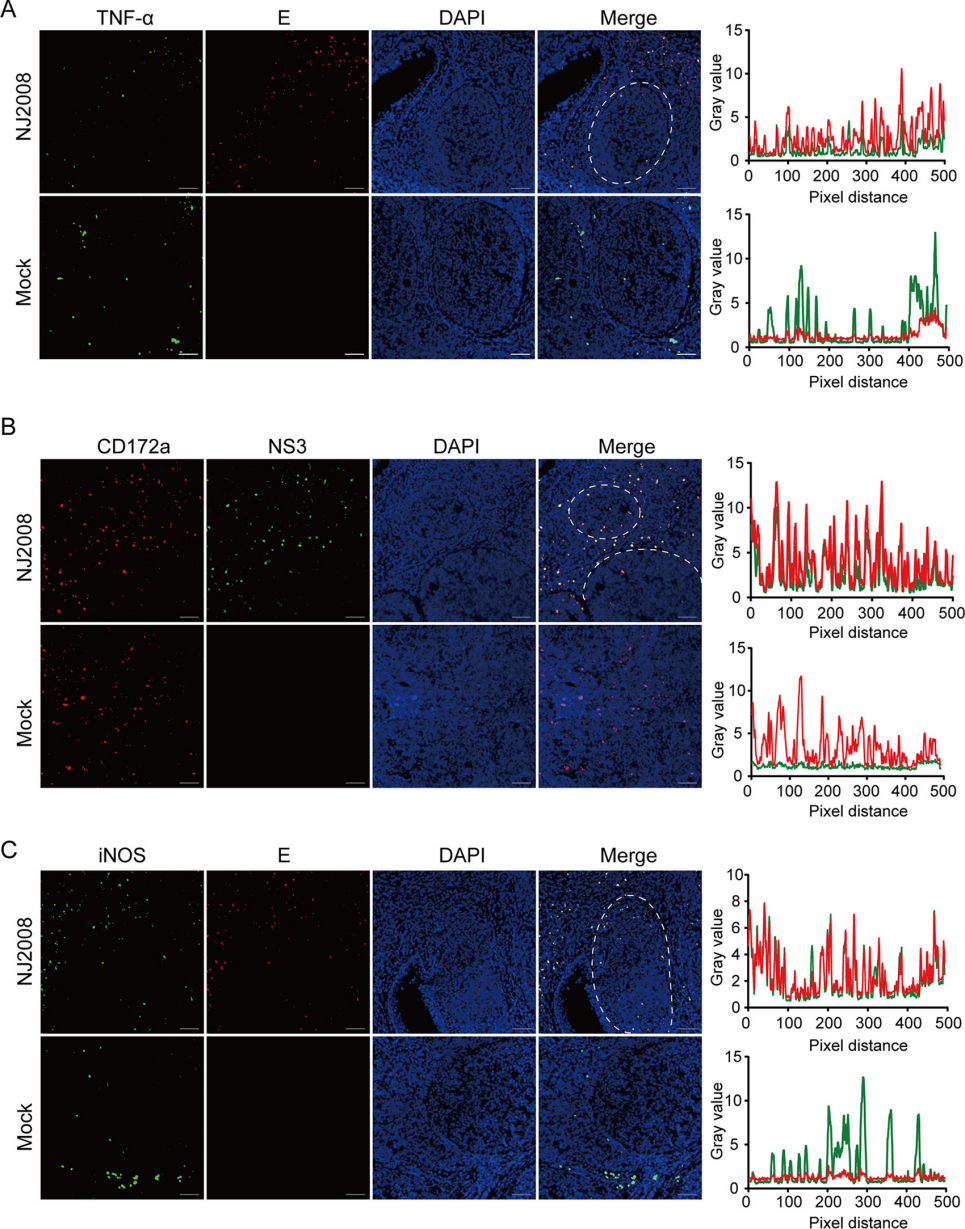
JEV antigen colocated with TNF-α-, CD172a-, and iNOS-positive cells. (A to C) Tonsils were sectioned into 5-μm slides and stained with antibodies against TNF-α (green) (A), CD172a (red) (B), iNOS (green) (C), E antigen (red), or NS3 antigen (green). Nuclei were stained with DAPI (blue). Magnification, ×10. The colocalization of cell markers and JEV antigen was analyzed with ImageJ software. Representative immunofluorescence images and colocalization line graphs of three independent experiments are shown.

DCs are specialized leukocytes that bridge the innate and adaptive immune systems. Several DC subsets belonging to distinct lineages and differing in their antigen uptake capacity have been characterized in lymphoid organs, mainly including classical DC (cDC) and plasmacytoid DC (pDC) (27, 28). To further determine the subtypes of JEV-infected DC, we stained tissue sections using the anti-tumor necrosis factor alpha (TNF-α) (predominantly expressed by cDC in pig tonsils) antibody. Immunofluorescence results showed relatively few TNF-α-positive cells in pig tonsils, but these cells were all infected by JEV (Fig. 9A). Unfortunately, we were not able to stain the pDC due to the lack of suitable antibodies. We could not determine whether pDCs are the target cells of JEV; however, cDC, as an important APC, can be infected by JEV. Two subpopulations of cDC (cDC1 and cDC2) have recently been described in pigs (29). CD172a, a cDC1-negative and cDC2-positive molecule, was selected to identify the DC subset tropism of JEV. Immunofluorescence results showed that high colocalization between CD172a and JEV was observed in the interfollicular region (Fig. 9B). Taken together, cDC2 cells are the target cells for JEV replication and may be a shelter for the persistence of JEV in pig tonsils.

### JEV NS1′ promoted virus infection in BMDCs and peritoneal macrophages *in vitro*.

**FIG 10:**
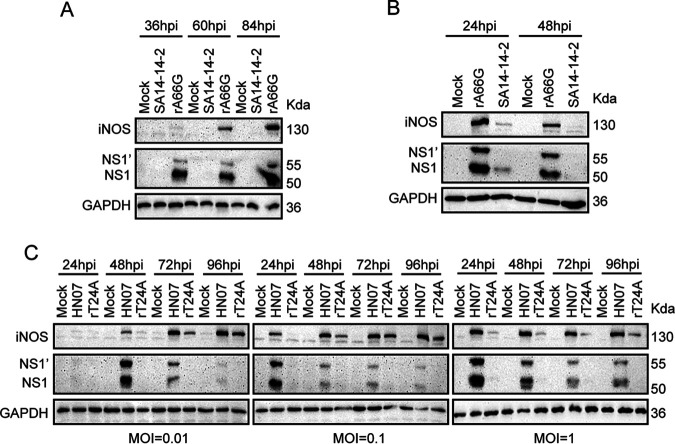
JEV infection significantly increases the expression of iNOS. (A and B) BMDCs (A) and PMs (B) were infected with JEV strain SA14-14-2 and rA66G mutant virus at an MOI of 1 for the indicated times. Immunoblot analysis of iNOS level in JEV infected cells. (C) The parent strain of rT24A mutant virus (not expressing NS1′) is JEV strain HEN0701. The 24th nucleotide of the *NS2A* gene was artificially mutated from T to A. BMDCs were infected with JEV strain HEN0701 and rT24A virus at a different MOI for the indicated times. JEV-induced iNOS activation was analyzed by immunoblot analysis. GAPDH, loading control. Data are representative of three separate biological replicates.

Additionally, inducible nitric oxide synthase (iNOS), which is mainly produced by cDC and macrophages, is one of the major mechanisms for killing pathogens *in vivo* (30). iNOS-producing cells were mainly distributed in the interfollicular region and colocalized with JEV (Fig. 9C). Interestingly, we found that iNOS was heavily activated after JEV infection. To further confirm this phenomenon, bone marrow-derived DCs (BMDCs) and peritoneal macrophages (PMs) were isolated from ICR mice and infected with SA14-14-2 or rA66G virus for the indicated times. rA66G virus infection led to a significant elevation of iNOS in BMDCs and PMs. However, no detectable or quite faint iNOS and NS1 bands were observed in the SA14-14-2 virus-infected groups (Fig. 10A and B), which indicated an antagonistic natural immune function of NS1′. Additionally, another virulent strain, HEN0701 (expressing NS1′), significantly activated iNOS after infection of BMDCs (Fig. 10C). In the rT24A mutant virus (not expressing NS1′)-infected group, no detectable NS1 bands were observed, but iNOS expression was still higher than in the mock group, which we recognize as a self-limiting infection of the virus (Fig. 10C). These results suggested that NS1′ promoted JEV infection in BMDCs and PMs, and the activation of iNOS was a host response to JEV infection. However, whether this virus-induced response is a host anti-infection mechanism or an immune escape strategy of JEV is still unknown.

## DISCUSSION

JEV NS1′ is known to suppress natural immunity and enhance the neuroinvasiveness and neurovirulence of virus in mice (18, 19). Pigs are important amplification hosts for JEV, and it is not clear whether NS1′ enhances JEV infection in pigs. In this study, we found that NS1′ significantly enhanced the infection of JEV in pigs, especially the tropism of CNS and tonsil tissues. Further studies showed that JEV mainly infected neurons in CNS tissues and caused irreversible neuronal damage, which is consistent with the mouse experiments (31). Importantly, DCs and macrophages are the main target cells of JEV infection in pig tonsils. JEV NS1′ promoted virus infection in DCs and macrophages. The reduction of MHC II and activation of iNOS caused by viral infection may create a beneficial environment for persistent JEV infection in pig tonsils. The results of our study suggest that JEV NS1′ enhances the viral infection of DCs and macrophages in pig tonsils.

The live-attenuated JEV vaccine (SA14-14-2 strain) was first approved for vaccination in 1989 in China and has exhibited an excellent safety profile and satisfactory efficacy (19, 32, 33). Despite the development of new JEV vaccines, SA14-14-2 remains the most widely used live attenuated vaccine (33). Pigs inoculated with JEV SA14-14-2 vaccine strain are transiently infected. After the inoculation of pigs with the live attenuated JEV vaccine, the virus cannot be detected in host organs and cannot cause long-term transmission (34). Animal experiments clearly showed that the single nucleotide mutation A66G in the *NS2A* gene increased the infection of JEV in pigs, especially in the CNS and tonsil tissues, which was consistent with the virulent strain NJ2008. Importantly, we found that the viral RNA was detected in oronasal swabs in the rA66G virus inoculation group. However, in the SA14-14-2 virus inoculation group, viral RNA was not detected, demonstrating that NS1′ may enhance the horizontal transmission of JEV. Vector-free transmission of JEV had been reported (7). Considering the high viral load and prolonged infection in the tonsils caused by JEV expressing NS1′, the lymphoid tissue of the oropharynx may be the source of the virus leading to the oronasal infection.

Although clinical symptoms were mild and no significant neurological deficits were observed, both the rA66G and NJ2008 virus inoculation groups showed neuronal damage, mainly manifesting as pathological changes of nonsuppurative meningoencephalomyelitis. Less pathological damage was seen in the cerebellum compared to the cerebral cortex and spinal cord (data not shown), which may be related to the lower viral load. Other studies did not show relatively low levels of JEV RNA in the cerebellum (6, 8, 35). We believe that the causes of this situation are small sample sizes and individual variations. Despite the small sample size, JEV RNA was detected in CNS tissues in all three pigs inoculated with rA66G virus, whereas none of the three pigs inoculated with SA14-14-2 virus had detectable virus. These results demonstrated that NS1′ could increase the neuroinvasiveness of JEV in pigs.

The most striking novel observations in terms of JEV tropism were found in the tonsils. High viral load persisted in tonsils despite the presence of neutralizing antibodies (7). An important finding of this study is that the target cells of JEV in tonsils are DCs and macrophages. In addition, DC subtype cDC2 cells are susceptible to JEV infection. DCs and macrophages are the main cells that produce type I interferon against viral infection (36). Proinflammatory cytokines are also highly expressed in JEV-infected tonsils. Mediators produced by DCs and macrophages can both enhance protective immune responses and promote infection, indicating duality in the response of APCs (37). Our results showed that iNOS was significantly elevated during infection. iNOS plays an important role in the fight against viral infection, but recent studies have shown that iNOS activation also suppresses natural immunity (38, 39). Additionally, hepatitis C virus (HCV) enhances expression of CXCR2 ligands in its host cell via the induction of epidermal growth factor (EGF) production, thereby recruiting neutrophile granulocytes, leading to chronic and persistent viral infection (40). Inflammatory factors such as iNOS can act in a paracrine fashion to recruit circulating monocytes (41). Circulating monocytes recruited to pig tonsils may serve as a source of cells for persistent JEV infection. The role of highly activated iNOS during JEV infection is unknown and requires further investigation. On the other hand, MHC II molecules are significantly reduced in JEV-infected tonsils, but the number of DCs and macrophages was unchanged. Thus, JEV infection significantly perturbs the transport of MHC II molecules and reduces the antigen cross-presentation capacity of DCs and macrophages. Induction of classical and nonclassical MHC I molecules due to infection with JEV was characterized in different cell lines (42, 43). JEV infection in the mouse brain can induce depletion of MHC I-positive cells in the thymus (44). However, there are few studies related to the depletion of MHC II molecules induced by JEV infection. Murine cytomegalovirus degrades MHC II molecules to colonize the salivary glands (45). Internalizing peptide-MHC II complexes are targeted for degradation by ubiquitination by the E3 ligase March-I in early endosomes (46). Whether JEV degrades MHC II molecules through these pathways is not known, and further study is needed.

Here, our results showed that NS1′ enhanced JEV infection in pig CNS and tonsil tissues. JEV primarily targeted DCs and macrophages for infection in pig tonsils. JEV NS1′ also promoted virus infection in BMDCs and PMs *in vitro*. Future work needs to focus on the mechanism by which NS1′ promotes JEV replication and persistent viral infection in pig tonsils.

## MATERIALS AND METHODS

### Cells and viruses.

Baby hamster kidney cells (BHK-21), adenocarcinomic human alveolar basal epithelial cells (A549), and pig kidney cells (PK-15) were cultured in Dulbecco’s modified Eagle’s medium (DMEM; 11965092; Gibco, Carlsbad, CA, USA). Mouse neuroblastoma cells (Neuro2a) and Aedes albopictus cells (C6/36) were cultured in RPMI 1640 medium (11875093; Gibco). All media were supplemented with 10% or 15% fetal bovine serum (FBS; 10100; GIBCO) and 1% penicillin-streptomycin. C6/36 cells were cultured at 28°C, while other cells were cultured at 37°C with 5% CO_2_. JEV NJ2008 (GenBank version no. GQ918133.2), SA14-14-2 (MK585066.1), HEN0701 (FJ495189.1), rT24A (a T-to-A mutation at nucleotide position 24 in the *NS2A* gene of the JEV HEN0701 strain), and rA66G (an A-to-G mutation at nucleotide position 66 in the *NS2A* gene of JEV strain SA14-14-2) were proliferated in C6/36 cells. The titers of the virus were titrated on BHK-21 cells by plaque assays.

The parental JEV (SA14-14-2) and NS1′ expression mutant virus (rA66G), or the parental JEV (HEN0701) and NS1′ defective virus (rT24A), were produced by BHK-21 cells with transcribed RNA from the full-length cDNA clones pWSK-SA14-14-2, pWSK-SA14-14-2-rA66G, pWSK-HEN0701, and pWSK-HEN0701-rT24A, respectively.

### Antibodies and reagents.

Mouse monoclonal antibodies against E, NS1, and NS1′ of JEV were generated in our laboratory. BALB/c mice were immunized with synthetic NS1 amino acid peptide or ΔNS1′ amino acid peptide (52 amino acids generated by −1PRF). Anti-NS1 or NS1′ monoclonal antibody was screened by the hybridoma cell fusion technique and indirect enzyme-linked immunosorbent assay (ELISA). Commercial antibodies used in this study are listed as follow: JEV NS3 protein rabbit polyclonal antibody (GTX125868; GeneTex), CD11b rabbit monoclonal antibody (ab133357; Abcam), CD163 rabbit monoclonal antibody (ab182422; Abcam), MHC II (also known as SLA) mouse monoclonal antibody (MCA2314GA; Bio-Rad), iNOS rabbit polyclonal antibody (NB300-605; Novus), CD172a mouse monoclonal antibody (NBP2-61014; Novus), TNF-α goat polyclonal antibody (AF690; R&D Systems), GAPDH mouse monoclonal antibody (AC033; ABclonal), goat anti-rat IgG Alexa Fluor 555 (A-21434; Invitrogen), goat anti-rabbit IgG Alexa Fluor 488 (A-11008; Invitrogen), goat anti-mouse IgG-horseradish peroxidase (HRP) (31430; Invitrogen), and goat anti-mouse IgG-HRP (31430; Invitrogen).

### Animal experiment.

A total of 10 commercial healthy 60-day-old large white pigs (5 castrated males and 5 females) born in November were purchased from local pig farmers. Each experimental group of pigs was housed in a separate room in a high-security isolation facility. Since detectable maternal antibodies disappeared in the majority of pigs by 4 to 6 months of age (47), antibody levels were detected by ELISA before JEV infection. All animals were allowed to adapt to the new environment.

Nine pigs were infected with 2 × 10^7^ TCID_50_ of JEV SA14-14-2 (*n* = 3), NJ2008 (*n* = 3), or rA66G (*n* = 3) mutant virus in a volume of 2 mL, giving one-half of the dose intravenously (i.v.) and the other half intradermally (i.d.) in the neck region. The remaining pig was injected with PBS as a control (*n* = 1). One pig from each JEV-inoculated group was painlessly executed at 3, 5, and 7 days postinfection, and the control pig was also executed at day 7. All animals underwent necropsy immediately after the painless execution.

Pigs were clinically examined daily, including temperature testing and nasal swabs collection until they were painlessly executed. At necropsy, tissues were collected to characterize the viral dissemination and tropism of JEV. All samples were stored at −80°C prior to subsequent experiments, including quantitative reverse transcriptase PCR (qRT-PCR), hematoxylin and eosin (H&E) staining, immunohistochemistry (IHC), and immunofluorescence (IF) as previously described (8).

### Western blotting.

Cells were infected with JEV for the indicated time and lysed in RIPA buffer (89900; Thermo Fisher) with protease and phosphatase inhibitors on ice for 30 min. Equal amounts of proteins were subjected to SDS-PAGE and transferred to the polyvinylidene difluoride (PVDF) membrane. The membranes were incubated with primary antibodies overnight at 4°C after being blocked with 5% nonfat milk for 2 h at room temperature (RT). For chemiluminescent readout, the membranes were incubated with horseradish peroxidase (HRP)-conjugated IgG secondary antibodies and exposed using BIO-RAD Clarity Western ECL substrate.

### Growth curve.

Virus titration was performed as previously described (48). Briefly, cells were incubated with JEV strain SA14-14-2 or rA66G virus at a multiplicity of infection (MOI) of 0.1 for 2 h. After 24, 48, 72, or 96 h, the supernatants were collected, and virus titers were determined by plaque assays.

### Quantitative reverse transcription polymerase chain reaction (qRT-PCR).

For RNA extraction, we used TRIzol (TaKaRa, 9109) for lysis of the homogenized tissue homogenates from different organs. The extracted RNA was introduced for reverse transcription using HiScript III RT SuperMix (R323-01; Vazyme) according to the instructions. qRT-PCR was performed with a SYBR green qPCR kit (711-02; Vazyme). JEV load was determined by detecting the viral envelope (E) gene and analyzed by the double standard curve method. For viral RNA detection, the gene expression levels were normalized to those of glyceraldehyde 3-phosphate dehydrogenase (GAPDH). Primer sequences are shown as follows: 5′-GGCAAACGACAAACCAACATT-3′ as the forward primer and 5′-ATCAGCTCGCTTCTCGTTGTG-3′ as the reverse primer for JEV E protein; 5′-GAGTCAACGGATTTGGTCGT-3′ as the forward primer and 5′-GACAAGCTTCCCGTTCTCAG-3′ as the reverse primer for GAPDH.

### Hematoxylin and eosin (H&E) staining.

Cerebrums and thalamus tissues were immersed in 4% paraformaldehyde, embedded in paraffin, sectioned at 5 μm, and stained with H&E. Slides were analyzed by an independent pathologist (Pathology Center, College of Veterinary Medicine, Nanjing Agricultural University) who was blinded to the experimental conditions.

### Immunohistochemistry (IHC).

Tissues were fixed in 4% paraformaldehyde and embedded in paraffin. Tissue sections were processed according to the instructions of the SP rabbit & mouse HRP kit (CWBIO; CW2069S). The slides were stained with JEV E, NS1′, or NS3 monoclonal antibodies and examined under a light microscope.

### Immunofluorescence (IF).

Tissue sections were permeabilized with 0.4% Triton X-100 for 30 min, washed three times with phosphate-buffered saline (PBS), and blocked with bovine serum albumin (BSA) for 1 h. Next, they were stained with the indicated primary antibodies at 4°C overnight, depending on the different experimental needs. These sections were washed with PBS and stained with fluorescence-conjugated secondary antibodies in the dark at 37°C for 2 h. Finally, the nucleus was stained with 4,6-diamidino-2-phenylindole (DAPI) at RT for 10 min.

### Isolation of mouse primary cells (PMs and BMDCs).

PMs were isolated as previously described (49), 4-week-old ICR mice were euthanized and disinfected with 75% ethanol, followed by intraperitoneal injection with 5 mL DMEM. After 5 min of peritoneal massage and 10 min of incubation, PMs were collected from the peritoneal cavity and centrifuged at 400 × *g* for 10 min. The cell precipitate was resuspended and cultured in DMEM containing 10% FBS. For BMDCs, bone marrow cells were cultured in 10 mL RPMI 1640 cell medium containing murine GM-CSF and recombinant mouse interleukin-4 (IL-4). On day 3 of culture, these cells were replaced with fresh granulocyte-macrophage colony-stimulating factor (GM-CSF) medium containing recombinant mouse IL-4. Adherent cells were transferred loosely to a fresh petri dish and cultured for an additional 4 days. For JEV infection, the PMs and BMDCs were infected with JEV at the indicated MOI.

### Statistical analysis.

Statistical analysis was performed an unpaired two-tailed *t* test and expressed as the mean ± standard deviation (SD). A *P* value of <0.05 was considered statistically significant. For the quantification method of colocalization, we imported the original images (merge) into ImageJ (software developed by the National Institutes of Health) and then performed Pearson coefficient analysis on the whole image to derive the values of red and green light, respectively. Finally, the values were generated as line graphs in GraphPad Prism 7.0 software.

### Pigs and mice.

All animal experiments were conducted under the guidelines of the regional Animal Ethics Committee and the rules for experimental animals of Nanjing Agricultural University.
